# Polymorphisms in the Gene Regions of the Adaptor Complex *LAMTOR2/LAMTOR3* and Their Association with Breast Cancer Risk

**DOI:** 10.1371/journal.pone.0053768

**Published:** 2013-01-16

**Authors:** Mariana E. De Araujo, Gertraud Erhart, Katharina Buck, Elisabeth Müller-Holzner, Michael Hubalek, Heidelinde Fiegl, Daniele Campa, Federico Canzian, Ursula Eilber, Jenny Chang-Claude, Stefan Coassin, Margot Haun, Lyudmyla Kedenko, Bernhard Paulweber, Roland Reitsamer, Irmgard Himmel, Dieter Flesch-Janys, Claudia Lamina, Florian Kronenberg, Lukas A. Huber, Anita Kloss-Brandstätter

**Affiliations:** 1 Division of Cell Biology, Innsbruck Medical University, Innsbruck, Austria; 2 Division of Genetic Epidemiology, Innsbruck Medical University, Innsbruck, Austria; 3 Division of Cancer Epidemiology, German Cancer Research Centre (DKFZ), Heidelberg, Germany; 4 Department of Obstetrics and Gynaecology, Innsbruck Medical University, Innsbruck, Austria; 5 Genomic Epidemiology Group, German Cancer Research Centre (DKFZ), Heidelberg, Germany; 6 First Department of Internal Medicine, Paracelsus Medical University, Salzburg, Austria; 7 Breast Center Salzburg, Paracelsus Medical University, Salzburg, Austria; 8 Department of Gynecology and Obstetrics, Hospital Meran, Meran, Italy; 9 Department of Medical Biometrics and Epidemiology, University Medical Center Hamburg-Eppendorf, Hamburg, Germany; IFOM, Fondazione Istituto FIRC di Oncologia Molecolare, Italy

## Abstract

**Background:**

The late endosomal LAMTOR complex serves as a convergence point for both the RAF/MEK/ERK and the PI3K/AKT/mTOR pathways. Interestingly, both of these signalling cascades play a significant role in the aetiology of breast cancer. Our aim was to address the possible role of genetic polymorphisms in *LAMTOR2* and *LAMTOR3* as genetic risk factors for breast cancer.

**Methodology/Results:**

We sequenced the exons and exon–intron boundaries of *LAMTOR2* (p14) and *LAMTOR3* (MP1) in 50 prospectively collected pairs of cancerous tissue and blood samples from breast cancer patients and compared their genetic variability. We found one single nucleotide polymorphism (SNP) in *LAMTOR2* (rs7541) and two SNPs in *LAMTOR3* (rs2298735 and rs148972953) in both tumour and blood samples, but no somatic mutations in cancerous tissues. In addition, we genotyped all three SNPs in 296 samples from the Risk Prediction of Breast Cancer Metastasis Study and found evidence of a genetic association between rs148972953 and oestrogen (ER) and progesterone receptor negative status (PR) (ER: OR = 3.60 (1.15–11.28); PR: OR = 4.27 (1.43–12.72)). However, when we additionally genotyped rs148972953 in the MARIE study including 2,715 breast cancer cases and 5,216 controls, we observed neither a difference in genotype frequencies between patients and controls nor was the SNP associated with ER or PR. Finally, all three SNPs were equally frequent in breast cancer samples and female participants (n = 640) of the population-based SAPHIR Study.

**Conclusions:**

The identified polymorphisms in *LAMTOR2* and *LAMTOR3* do not seem to play a relevant role in breast cancer. Our work does not exclude a role of other not yet identified SNPs or that the here annotated polymorphism may in fact play a relevant role in other diseases. Our results underscore the importance of replication in association studies.

## Introduction

Scaffold proteins were originally identified in yeast and are now recognized to contribute to the specificity of MEK/ERK pathways in mammalian cells. LAMTOR3 (MP1) was identified in a yeast two-hybrid screen as a specific binding partner of MEK1 [1], that is recruited to late endosomes by the adaptor protein LAMTOR2 (p14) [2]. MP1 and p14 are structurally almost identical and form a very stable heterodimeric complex that is required for ERK activation on endosomes [3], [4]. Using conditional gene disruption of p14, it was previously shown that the p14/MP1-MEK1 signalling complex regulates late endosomal traffic, EGFR degradation and cellular proliferation [5]. This function is essential for early embryogenesis and during tissue homeostasis as revealed by epidermis-specific deletion of p14 [5]. Taken together, endosomal p14/MP1-MEK1 signalling has a specific and essential function *in vivo*: it contributes to the regulation of late endosomal traffic by extra-cellular signals, that in turn is required to maintain tissue homeostasis.

**Figure 1:**
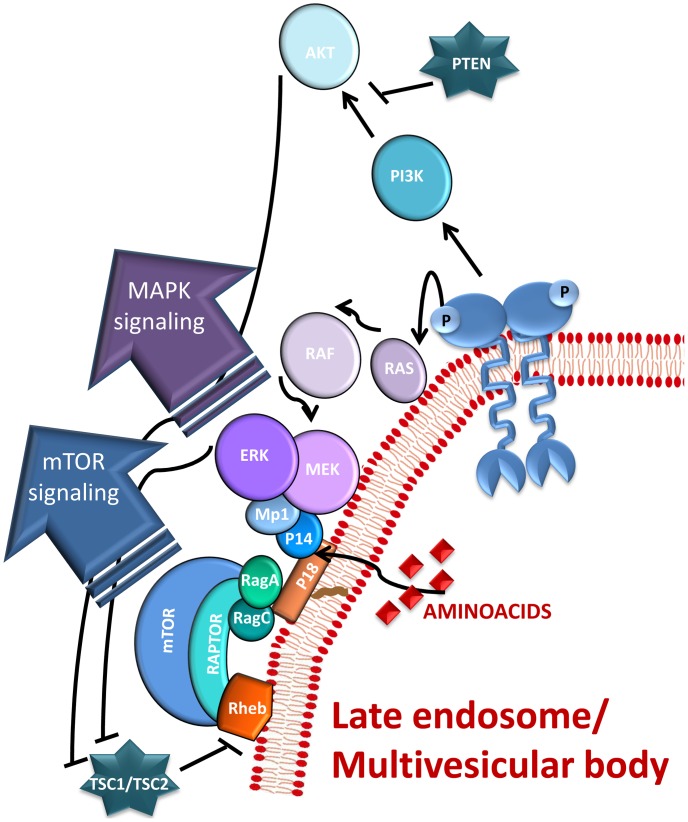
The LAMTOR complex as a convergence point of MAPK and mTORC1 pathways (Schematic overview). Internalized activated receptors keep their ability to signal while they traffic through the endocitic compartment. The arrival at late endosomes/multivesicular bodies of the activated receptor and the detection of aminoacids in the lumen of the organelle, trigger a cascade of phosphorylation events leading to the local activation of both mTORC1 and ERK1/2. The p18/Mp1/p14 complex, also known as the LAMTOR complex was shown to function as a convergence point for both pathways. Both signaling cascades were simplified for didactic reasons.

Anchorage of the p14/MP1/MAP kinase pathway to late endosomes is mediated by a small lipid raft adaptor called LAMTOR1 (p18), which directly binds endosomal lipids [6]. The trimeric complex p18/p14/MP1 was recently shown to mediate the translocation of mTORC1 to lysosomal membranes, a critical event in amino acid signalling to mTORC1 [7]. The mTORC1 kinase promotes growth in response to growth factors, energy levels, and amino acids, and its activity is often deregulated in disease conditions. In brief, these data highlight the role of the endosomal scaffold complex p18/p14/Mp1 as a convergence point of signalling pathways controlling proliferation (Figure 1).

Progression, proliferation, and hormone independent growth of breast cancer cells is dependent on MAP kinase (ERK) activity [8]. Furthermore, there are consistent reports that primary breast tumors and tissues display elevated expression and activity levels of ERK [9]. In most breast cancers, ERK hyper-activation is due to over-expression and/or constitutive activation of upstream regulators like ErbB2, c-Src or GrB2 [10], [11], [12]. Sustained ERK1/2 signalling in cooperation with transforming growth factor ß (TGFß) activation promote epithelial mesenchymal transition (EMT) and increase invasiveness and metastatic potential of cultured mammary epithelial cells [13]. In brief, ERK activation induces matrix metalloproteinases that degrade collagen, it can inactivate integrin-meditated cell adhesion and activates the myosin light chain thereby eliciting cell migration. Importantly, the Ets-1 transcription factors are key ERK substrates that have been shown to induce EMT and invasiveness in a number of breast cancer cell lines [14], [15]. Several anti-cancer therapies result in the induction of the Raf/MEK/ERK pathway that may provide a survival signal for the tumour, thereby potentiating resistance to treatment. For example, ERK1/2 phosphorylates Ser118 of oestrogen receptor alpha, providing a mechanism by which the oestrogen receptor can be activated in a ligand independent manner [16].

The PI3K/AKT signalling cascade is another major player in cancer progression. Mutations in the PI3K subunit p110 are found in roughly 25% of breast cancers [17], [18]. Furthermore, loss of the tumour suppressor protein PTEN has been directly implicated in hereditary breast cancer [19]. Both the RAF/MEK/ERK and the PI3K/AKT pathways modulate several key apoptotic players thereby transducing a survival signal. The ERK pathway regulates BcL-2, Bad, Mcl-1, Bim, Survivin and Caspase 9 [20], [21], [22]. The PI3K/AKT pathway phosphorylates Bim, Bad, XIAP and p21 [23], [24], [25].

It has been recently shown that treatment of breast cancer cells with MEK or mTOR inhibitors and either Doxorubicin or Tamoxifen results in a synergistic response that highlights the advantages of combining classical chemotherapy with targeted adjuvant treatments [19]. For example, Tamoxifen resistant breast cancer cells with overexpressed/activated v-akt murine thymoma viral oncogene homolog (AKT) or lack of phosphatase and tensin homologue (PTEN) may benefit from Rapamycin treatment, a highly specific mTOR inhibitor. In addition, the complementary use of RAF/MEK/ERK inhibitors may provide an added value in the treatment of this type of tumours since ERK is known to phosphorylate TSC2. The TSC1/TSC2 complex, also known as the tuberous sclerosis complex, controls the small G-protein Rheb through its GAP activity, thereby functioning as a critical negative regulator of mTORC1.

*Interestingly, in 2007,* Conrad *et al.* submitted a patent on *LAMTOR3* (Mp1) as a diagnostic and therapeutic target for breast cancer treatment and prevention (United States patent Application No. US 2007/0172843 A1; International publication nr. W = 2007/033118 A2). In addition, a recent publication from the same group reports that *LAMTOR3* (Mp1) is required for the survival of estrogen receptor positive breast cancer cell lines [26].

Taking into consideration the above report and recent findings identifying the LAMTOR complex as a convergence point for both the ERK and mTORC1 pathways, we aimed to investigate the potential role of mutations in *LAMTOR3* and *LAMTOR2* in the aetiology of breast cancer.

## Materials and Methods

### Ethics Statement

This study was approved by the ethics committee of the Innsbruck Medical University (study code UN3377).

### Patient Characteristics at the Screening Stage

For mutation screening, tissue samples of 50 consecutive breast cancer patients were prospectively collected at the Innsbruck Medical University starting in July 2009. Patients aged 18 or older, who had signed an informed consent, were consecutively included in the study. The following clinical parameters were collected: age; menopausal status; tumour histology; tumour size; tumour grade; lymph node status; oestrogen receptor status; progesterone receptor status; HER2 (human epidermal growth factor receptor 2) status; and presence of metastasis.

### Sequencing of Exons in *LAMTOR2* (p14) and *LAMTOR3* (MP1)

Genomic DNA was extracted from frozen tumour tissue or from peripheral blood collected on EDTA on a BioRobot EZ1 advanced Workstation with the EZ1 DNA tissue or blood kit (QIAGEN, Hilden, Germany) and quantified with a NanoDrop spectrophotometer (Thermo Fisher Scientific Inc., Waltham, MA). Amplification and sequencing primes were designed with Visual OMP (DNA Software, Inc., Ann Arbor, MI).

All four exons of the *LAMTOR2* gene (following the nomenclature of transcript ENST00000368305, Ensembl Release 52; www.ensembl.org) were amplified in 2 PCR reactions and sequenced with 8 primers (Table S1). An overview of the amplification and sequencing strategy of the exons within *LAMTOR2* is given in Figure S1.

Five out of seven exons of the *LAMTOR3* gene (following the nomenclature of transcript ENST00000226522, Ensembl Release 52; www.ensembl.org) were amplified in 4 PCR reactions and sequenced with 14 primers (Table S2). The genomic region including Exon 1 and Exon 2 could be amplified in one PCR reaction. Exon 3, Exon 4, Exon 5 and Exon 6 were each very short and had long intronic stretches between each other, so that none of these exons could be targeted with another PCR reaction. Exon 3 codes for the first alpha helix of LAMTOR3, is only 35bp long, lacks putative protein binding sites and shows low amino acid conservation [4]; therefore, it was not of particular structural interest and was excluded from sequencing. Exon 6 codes for two of the central β-strands of LAMTOR3. Non-synonymous mutations in Exon 6 (64bp) are assumed to have two possible consequences: if the properties of the amino acids were maintained, LAMTOR3 would fold correctly and the protein would be as functional as the wild type. If the amino acid properties significantly changed, the correct assembly of the central β-sheet would be impaired, thereby completely abolishing LAMTOR3 function, with the consequence that LAMTOR3 molecules would be degraded by quality control mechanisms. If such mutations existed, they would lead to embryonic lethality as was observed in LAMTOR2 knockout mice [5]. Therefore, we also excluded Exon 6 from sequencing. An overview of the amplification and sequencing strategy of the selected exons within *LAMTOR3* is given in Figure S2.

All fragments except for MP1-1 were amplified in a total reaction volume of 25 µl, containing 70–130 ng of DNA, 0.5 µl of Herculase II Fusion DNA Polymerase (Stratagene, La Jolla, USA), 5 µl of PCR reaction buffer, 250 µM each dNTP, and 0.25 µM each primer. The reaction cocktails were heated to 95°C (2 min) and then put through 35 amplification cycles: 95°C for 20 s, 55°C for 20 s, and 72°C for 60 sec and a final extension phase at 72°C for 10 min. Fragment MP1-1 was amplified in a total reaction volume of 25 µl, containing 60 ng of DNA, 0.5 µl of KAPA HiFi HotStart DNA Polymerase (KAPA Biosystems, Boston, USA), 5 µl of 5X KAPA HiFi GC Buffer, 0.5 µl KAPA dNTP Mix (10 mM each dNTP), and 0.3 µM each primer. The reaction cocktails were heated to 95°C (5 min) and then put through 30 amplification cycles: 98°C for 20 s, 56.7°C for 15 s, and 72°C for 45 sec and a final extension phase at 72°C for 5 min.

PCR products were purified using QIAvac vacuum manifolds (QIAGEN) and eluted in 50µl distilled water according to the manufacturer’s protocol. For cycle sequencing, 3 µl of purified PCR product were combined with the sequencing master mix (containing 2 µl BigDye Terminator v1.1 Cycle Sequencing RR mix [AB], 2 µl Sequencing Buffer [AB], 0.3 µM primer, and distilled water up to 10 µl) and cycled (after a first denaturation step of 96°C, 2 min) for 30 cycles of 30 s at 96°C, 20 s at 55°C, and 1 min at 60°C. Purification of cycle-sequencing products with Sephadex (GE Healthcare) was performed according to the procedure described in Brandstätter et al. [27].

Electrophoretic separation was carried out on an ABI3130*xl* capillary sequencer with POP-7 and a 36 cm capillary array.

### Risk Prediction of Breast Cancer Metastasis Study

For studying the effects of the identified variants in a breast cancer sample, one single nucleotide polymorphism (SNP) in *LAMTOR2* (rs7541) and two SNPs in *LAMTOR3* (rs2298735 and rs148972953) were genotyped using a TaqMan® SNP Genotyping Assay on a 7900HT Fast Real-Time PCR System (Applied Biosystems, Foster City, CA) in 296 samples of the Risk Prediction of Breast Cancer Metastasis Study. This study is a multicenter study including prospectively collected breast cancer samples from the cities Innsbruck (Austria), Salzburg (Austria) and Meran (Italy). Patients aged 18 or older, who had signed an informed consent, were consecutively included in the study. Detailed information on tumour characteristics (e.g., hormone receptor status) and treatment (e.g. chemotherapy, endocrine therapy, and radiotherapy) were collected using clinical and pathological records. The Risk Prediction of Breast Cancer Metastasis Study was approved by the ethics committee of the Innsbruck Medical University (study code AM330a). The genotyping success rates were 97.3% for rs7541, 99.3% for rs2298735, and 97.3% for rs148972953.

### Replication: MARIE Study

For a more detailed analysis, the SNP rs148972953 was genotyped using a TaqMan® SNP Genotyping Assay on a 7900HT Fast Real-Time PCR System (Applied Biosystems, Foster City, CA) in 7,931 samples of the MARIE Study. The MARIE (Mammary carcinoma Risk factor Investigation) study population comprises breast cancer patients who participated in a population-based case-control study conducted in two German study regions (Hamburg and Rhine-Neckar-Karlsruhe) [28]. The study was approved by the ethics committees of the University of Heidelberg and the University of Hamburg. Patients were eligible if they had a histologically confirmed primary invasive (stage I-IV) or in situ breast tumour, were 50 to 74 years old, resident in the study region, and German-speaking. Of the 6,114 eligible patients, 3,919 (64.1%) participated in the study. Detailed information on tumour characteristics (e.g., hormone receptor status) and treatment (e.g. chemotherapy, endocrine therapy, and radiotherapy) were collected using clinical and pathological records/attending physicians. Two controls per case were randomly selected from population registries frequency-matched by year of birth and study region. Of the 17,093 eligible controls, 7,421 (43.3%) participated in the study. For the present analyses, 2,767 cases and 5,324 controls were included, excluding subjects without blood samples and genotype information. Information on socio-economic and lifestyle factors was collected at baseline by means of a personal interview. An overview of all three breast cancer populations is given in Table 1. The genotyping success rates were 98.1% for cases, and 98.0% for controls. The fraction of samples that were genotyped twice for quality assurance were 9.5%, the genotyping discordance rate was 0%. Therefore genotype information was available for 2,715 cases and 5,216 controls.

**Table 1:** Characteristics of the breast cancer studies.

	Sequencing	RPBCMS	MARIE cases
n	50	296	2,715
Age at diagnosis (years)	58.3±13.4	59.7±13.3	62.4±6.1
Percentage of premenopausal women	31.3%	34.3%	9.2%
Histology			
IDC	74.0%	75.0%	66.3%
ILC	16.0%	15.5%	19.9%
DCIS		1.7%	6.3%
other	10.0%	7.8%	7.3%
Tumour size			
with the diameter lessthan 2 cm	54.0%	58.0%	51.5%
with the diameter morethan 2 cm	46.0%	42.0%	42.2%
Tumour grade			
I	4.0%	8.0%	17.4%
II	78.0%	69.5%	48.1%
III	18.0%	22.5%	28.1%
Oestrogen receptor status			
positive	84.0%	86.3%	79.0%
negative	16.0%	13.7%	21.0%
Progesterone receptor status			
positive	76.0%	75.2%	67.0%
negative	24.0%	24.8%	33.0%

Notes: the age at diagnosis is indicated as mean value ± standard deviation.

Abbreviations:

IDC… infiltrative ductal carcinoma.

ILC… infiltrative lobular carcinoma.

DCIS… ductal carcinoma in situ.

RPBCMS… Risk Prediction of Breast Cancer Metastasis Study.

### SNP Frequencies in a Healthy Working Population: SAPHIR Study

For studying the frequency of the identified variants in a healthy working population, one SNP in *LAMTOR2* (rs7541) and two SNPs in *LAMTOR3* (rs2298735 and rs148972953) were genotyped using a TaqMan® SNP Genotyping Assay on a 7900HT Fast Real-Time PCR System (Applied Biosystems, Foster City, CA) in female participants of the SAPHIR Study. The Salzburg Atherosclerosis Prevention Program in Subjects at High Individual Risk (SAPHIR) is an observational study conducted in the years 1999–2002 involving 1,770 healthy unrelated subjects: 663 females from 39 to 67 years of age and 1,107 males from 39 to 66 years of age [29]. Study participants were recruited by health screening programs in large companies in and around the city of Salzburg. DNA was available for 640 female samples. The fraction of samples that were genotyped twice for quality assurance were 4%, the genotyping discordance rate was 0%. The genotyping success rates were 99.1% for rs7541, 97.3% for rs2298735, and 97.2% for rs148972953.

### Statistical and Bioinformatic Analysis

The genotype distribution was used to calculate minor allele frequencies and deviations from Hardy-Weinberg equilibrium were evaluated using a Chi-square test. Due to the low minor allele frequency of rs148972953, heterozygous and homozygous minor allele carriers were assessed combined in comparison to major allele carriers. Differences in allele frequencies between carriers and non-carriers of rs148972953 by oestrogen receptor status, progesterone receptor status, metastasis at baseline and metastasis after treatment were calculated by using a Chi-squared test. Odds ratios with a 95% confidence interval were calculated for estimating the relationship between rs148972953 and oestrogen receptor status (ER), progesterone receptor status (PR) and metastasis [30]. Statistical analyses were performed with SPSS (version 19), R (version 2.15.0) and SAS (version 9.2).

Following functional considerations based on the SNP position, the potential effects of the three SNPs were investigated using selected bioinformatic applications [31]. Especially, the SNPs were checked for exonic splicing regulators (ESRs) with F-SNP [32] and miRNA binding sites with Patrocles [33] and mirRBase [34].

## Results

### Search for Genetic Variability in *LAMTOR2* and *LAMTOR3*

Sequencing of the exons and exon-intron boundaries of both *LAMTOR2* and *LAMTOR3* revealed three SNPs to occur in a sequencing sample of 50 breast cancer patients, but no novel mutations. One SNP was found in *LAMTOR2* (rs7541) and two SNPs within *LAMTOR3* (rs2298735 and rs148972953) (Table 2). Our hypothesis that *LAMTOR2* and *LAMTOR3* gene regions would harbour somatic mutations in tumour tissue was not confirmed; all SNPs that were found were present in both benign (DNA derived from whole blood) and cancerous tissue.

**Table 2:** Genotype frequencies of the analyzed SNPs by study population.

	*LAMTOR2*	*LAMTOR3*	
	rs7541	rs2298735	rs148972953
Chromosome: Base pair position	1∶156,025,096	4∶100,815,617	4∶100,802,946
Located in:	Exon 2	5′UTR	3′UTR
Ancestral/derived allele	C/T	T/G	A/G
SNP effect	Synonym	–	–
GD: AA/Aa/aa: Sequencing	66.0/28.0/6.0%	28.0/40.0/32.0%	92.0/8.0/0.0%
GD: AA/Aa/aa: RPBCMS	75.4/22.2/2.4%	39.1/45.2/15.7%	96.5/3.5/0.0%
GD: AA/Aa/aa: SAPHIR women	70.3/27.0/2.7%	39.5/44.5/16.0%	96.0/3.9/0.1%
GD: AA/Aa/aa: MARIE cases	n.a.	n.a.	97.1/2.9/0.0%
GD: AA/Aa/aa: MARIE controls	n.a.	n.a.	97.3/2.7/0.0%
MAF: Sequencing (n = 50)	20.0%	52.0%	4.0%
MAF: RPBCMS (n = 296)	13.5%	38.3%	1.7%
MAF: SAPHIR women (n = 640)	16.2%	38.2%	2.1%
MAF: MARIE cases (n = 2,715)	n.a.	n.a.	1.5%
MAF: MARIE controls (n = 5,216)	n.a.	n.a.	1.4%
HWE p-value: Sequencing	0.376	0.159	0.768
HWE p-value: RPBCMS	0.387	0.466	0.764
HWE p-value: SAPHIR women	0.900	0.140	0.153
HWE p-value: MARIE cases	n.a.	n.a.	0.435
HWE p-value: MARIE controls	n.a.	n.a.	0.316

**Notes:**

Call rates in all study populations were above 98%.

MAF… Minor allele frequency.

HWE… p-value for test for Hardy-Weinberg-Equilibrium (Chi-Square test).

GD… Genotype distribution (in %).

RPBCMS… Risk Prediction of Breast Cancer Metastasis Study.

### Bioinformatic Analysis

Bioinformatic analysis indicated that the synonymous SNP rs7541 in exon 2 of *LAMTOR2* could possibly affect splicing regulation (predicted by both F-SNP [32] and PupaSuite [35]) by deletion of a splicing silencer element. However, none of the annotated NCBI transcripts actually shows splicing in this region. The SNP rs2298735, which is located in the 5′UTR of *LAMTOR3*, had a very low functional score in F-SNP, and we also did not find transcription factor binding sites (as predicted with FASTSNP [36]).

Interestingly however, the rare allele of SNP rs148972953 in the 3′UTR of *LAMTOR3* abolishes a putative binding site for the miRNA mir-126* (“TAA**T**AATA”) (as analyzed with Patrocles [33] and miRBase [34]). mir-126* and its complement mir-126, which are encoded by intron 7 of the egfl7 gene, have been reported to impair cancer progression through signalling pathways that control tumour cell proliferation, migration, invasion, and survival in a wide variety of cancers [37], [38], [39], [40], [41], [42], [43], especially in breast cancer [44], [45].

### Genetic Association Studies and Comparison with Healthy Controls

As our sequencing sample was relatively small (n = 50), and as the SNP rs148972953 in *LAMTOR3* had a minor allele frequency (MAF) of 4%, we investigated whether a healthy working population sample would show different genotype distributions than the breast cancer sample. Therefore, we genotyped all three SNPs in female participants of the SAPHIR population (n = 640). The allele and genotype frequencies in SAPHIR women were similar to those in our sequencing sample (p>0.05; Table 2).

For the analysis of genetic association, we combined the samples for sequencing and those in the Risk Prediction of Breast Cancer Metastasis Study since they were collected at the same University Clinic in the same manner. In the combined breast cancer sample, rs148972953 in *LAMTOR3* was found to be strongly associated with negative progesterone receptor status (OR = 4.27 (1.43–12.72); Table 3) and with negative oestrogen receptor status of the tumour (OR = 3.60 (1.15–11.28); Table 3).

**Table 3:** Genetic association of rs148972953 with tumour characteristics (oestrogen and progesterone receptor status and metastases).

		Oestrogen receptor		Progesterone receptor	
Study population	rs148972953	positive	negative	positive	negative
*Sequencing +*	Wild-type (GG)	266	41	234	73
*RPBCMS*	Carrier (AG or AA)	9	5	6	8
*Combined*	p-value	0.035		0.006	
*(n = 321)*	OR (95% CI)	1	3.60 (1.15–11.28)	1	4.27 (1.43–12.72)
*MARIE cases*	Wild-type (GG)	1919	504	1610	809
*(n = 2,499)*	Carrier (AG or AA)	58	18	51	25
	p-value	0.543		0.920	
	OR (95% CI)		1.18 (0.69–2.02)	1	0.97 (0.60–1.58)

**Notes:**

Due to the low minor allele frequency of rs148972953, heterozygous and homozygous mutation allele carriers were assessed combined in comparison to wild-type allele carriers assuming a dominant mode of inheritance.

For this analysis, only samples with complete information on ER, PR and an rs148972953 genotype were taken into consideration.

OR … odds ratio.

95% CI … 95% confidence interval.

RPBCMS… Risk Prediction of Breast Cancer Metastasis Study.

### Replication in the MARIE Study

Encouraged by the findings from the combined breast cancer sample and the bioinformatic analyses we performed a replication in the MARIE Study. The minor allele of rs148972953 was not found to show differential association by oestrogen receptor or progesterone receptor status (ER: OR = 1.18 (0.69–2.02); PR: OR = 0.97 (0.60–1.58); Table 3). In addition, cases and controls of the MARIE Study did not differ in the genotype frequency of rs148972953 (Chi-Square Test; p = 0.60).

Based on the results of the bioinformatics analysis, we hypothesized that rs148972953 could be associated with tumour metastasis. However, our assumption was not supported in the MARIE Study. Compared to patients carrying the wildtype of rs148972953 polymorphism, carriers of the minor allele of rs148972953 were neither more likely to have metastasis at baseline (OR = 1.76 (0.62–4.96)) nor were they at higher risk of distant disease-free survival (HR = 0.96 (0.75–1.23)) (Table 4). The genetic status at rs148972953 did not modify the response to primary hormone therapy (overall survival: HR = 0.83 (0.35–2.07); distant disease free survival: HR = 0.96 (0.72–1.29); recurrence-free survival: HR = 1.01 (0.76–1.35)). Carrying the minor allele of rs148972953 was also not associated with the time until tumour relapse (Mann-Whitney test; p = 0.947).

**Table 4:** Genetic association of rs148972953 with risk of having metastasis in MARIE breast cancer cases.

		Wild-type(GG)	Carrier(AG or AA)
Metastasis at	Yes	73	4
baseline	No	2221	69
	p-value	0.276	
	OR (95% CI)	1	1.76 (0.62–4.96)
Metastasis after	Yes	203	8
primary treatment	No	2252	66
	p-value	0.435	
	OR (95% CI)	1	1.34 (0.63–2.84)

**Notes:**

The analysis was only performed in breast cancer cases of the MARIE Study. At baseline, 2367 patients suffered from a primary invasive breast cancer and 159 patients suffered from in situ breast cancer; those 159 patients were excluded for this analysis. In the Risk Prediction of Breast Cancer Metastasis Study, information on metastasis was either missing in most patients or the follow-up time was too short for the development of metastasis after primary treatment.

OR … odds ratio.

95% CI … 95% confidence interval.

## Discussion

Bioinformatic analysis of high-throughput cancer microarrays available on the public domain Oncomine [46], [47] revealed that *LAMTOR2* (p14) is significantly up-regulated in breast cancer cells. Interestingly, two independent publications also reported an up-regulation of p14 in invasive ductal breast carcinomas [46], [48]. In addition, *LAMTOR3* (Mp1) was also found to be up-regulated in invasive ductal breast carcinomas [49]. Invasive ductal breast carcinoma is the most common type of breast cancer, comprising 70% to 80% of all cases. It commonly spreads to the regional lymph nodes and carries a poor prognosis. Interestingly, *LAMTOR3* (Mp1) was also reported to exhibit reduced levels in the stroma of invasive breast carcinoma [50]. The apparently opposing observations may indicate that Mp1 is differentially regulated in stroma and tumour cells. In addition, a recent publication reported that *LAMTOR3* (Mp1) was required for the survival of oestrogen receptor positive breast cancer cell lines [26]. Taking into consideration the above data, we hypothesized that LAMTOR components may indeed play a relevant role in breast cancer progression and are not simply innocent bystanders. Changes in intracellular protein levels can depend on variation in the rates of transcription, translation and degradation. In this project, we searched for mutations in the genomic regions of *LAMTOR2* and *LAMTOR3* that could contribute to the aetiology of breast cancer by altering any of the above mentioned processes.

We initially hypothesized that somatic, cancer-tissue-specific mutations in *LAMTOR2* and *LAMTOR3* could be associated with breast cancer progression and metastasis. We additionally expected that several rare instead of common variants would be detected and those rare variants were not expected to be already listed in databases. Therefore, we applied a discovery stage by sequencing the exons of *LAMTOR2* and *LAMTOR3* in 50 cancerous tissues and the corresponding peripheral leukocyte DNA and observed no differences in the identified mutations between the two tissues. The identified mutations were investigated in two different case samples (Risk Prediction of Breast Cancer Metastasis Study and MARIE cases) as well as two different control samples (SAPHIR women and MARIE controls).

Despite the promising results in the initial study, the replication study failed to support an association between the SNP rs148972953 in *LAMTOR*3 and both oestrogen and progesterone receptor status and metastasis. The present work does not exclude that the observed polymorphisms may play a role in other disease contexts or that other not yet identified polymorphisms in *LAMTOR2* and *LAMTOR3* may in fact contribute to breast cancer aetiology. We would also like to emphasize that genetic variation is not the only factor contributing to disease progression. Many regulatory aspects, in particular those controlling protein stability, posttranslational modifications and association with binding partners, play a fundamental role in determining how much and how active a protein actually is. As discussed before, previous publications identified the LAMTOR complex as a convergence point of key signalling pathways: MAPK and mTOR. Due to the well established role of both signalling cascades in breast cancer progression, and the recent implication of *LAMTOR3* in oestrogen receptor positive breast cancer, we leave open the possibility of therapeutically targeting the complex as previously proposed by others [26].

Although the LAMTOR complex was independently demonstrated to be involved in breast cancer [46], [48], [49], [50], neither *LAMTOR2* nor *LAMTOR3* came up as hits in genome-wide association studies (GWAS) with any kind of disease or trait as indexed by the catalogue of published GWAS [51]. One reason for this could be that some gene regions are poorly covered by recent SNP arrays; e.g. rs148972953 is currently neither available on any commercial genotyping array nor can it be imputed based on HapMap data. However, the region 4q23, in which *LAMTOR3* is located, showed signals of associations with upper aerodigestive tract cancers [52] and oesophageal cancer [53] indicating that the region *per se* is interesting for cancer. Therefore, future studies including a dense map of SNPs from the LAMTOR complex including the regulatory regions might reveal implications of genetic associations of *LAMTOR2* and *LAMTOR3* with breast cancer.

The present study constitutes a good example for the necessity and importance of replication in genetic association studies. Small cohorts intrinsically increase the number of false positives that are accepted. Replication in larger populations is therefore fundamental to separate the wheat from the chaff.

## Supporting Information

Figure S1 Amplification and sequencing strategy of *LAMTOR2.*
(DOC)

Figure S2 Amplification and sequencing strategy of *LAMTOR3.*
(DOC)

Table S1 Primers used for amplification and sequencing of *LAMTOR2.*
(DOC)

Table S2 Primers used for amplification and sequencing of *LAMTOR3.*
(DOC)
