# Supplementary figures and images for: Polymorphisms in the Gene Regions of the Adaptor Complex LAMTOR2/LAMTOR3 and Their Association with Breast Cancer Risk
Source: PLoS One. 2013 Jan 16;8(1):e53768. doi: 10.1371/journal.pone.0053768 (PMC3547070; doi:10.1371/journal.pone.0053768)

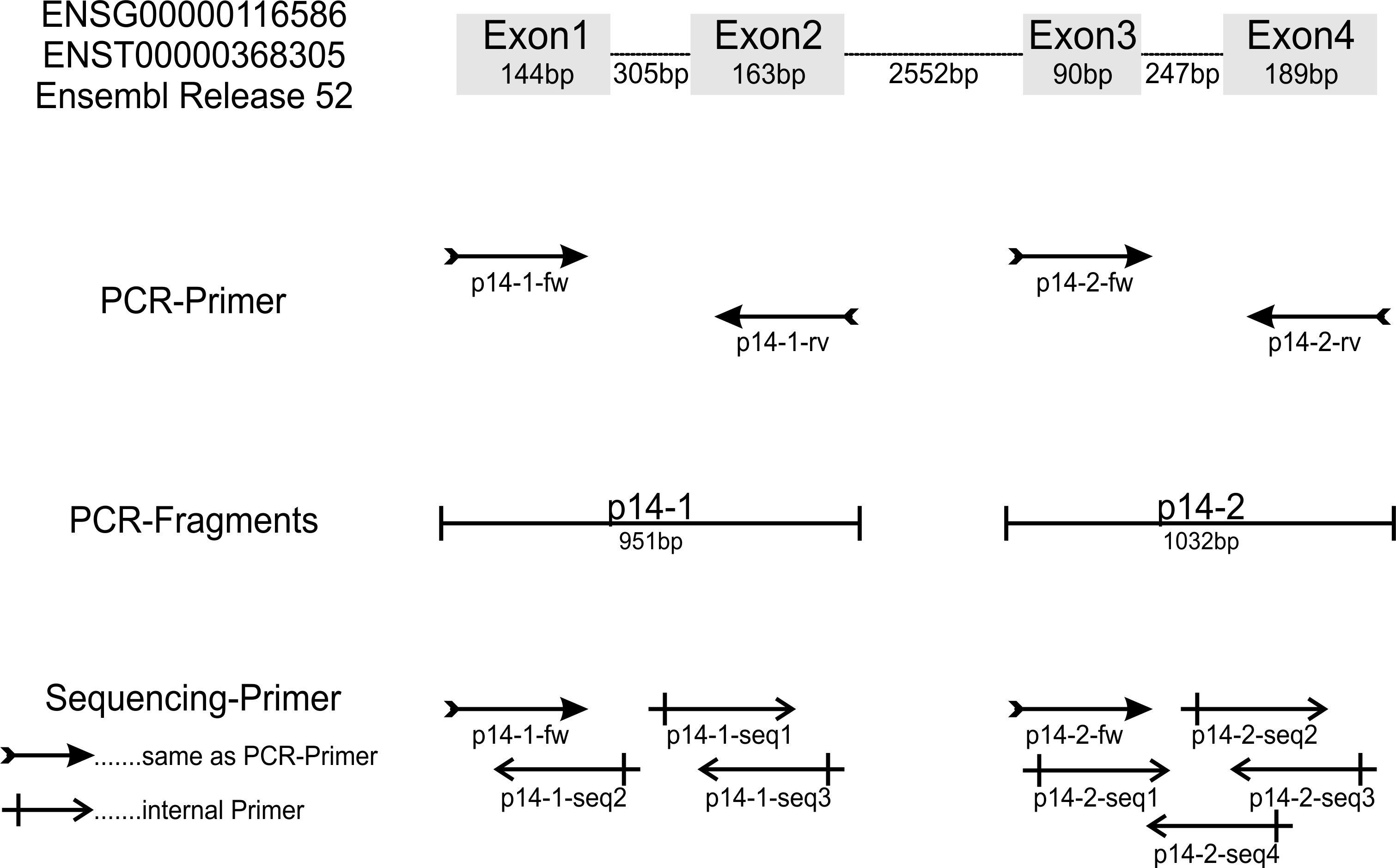
**Supplementary Figure S1** Amplification and sequencing strategy of *LAMTOR2*

Supplement: Figure S1 — Amplification and sequencing strategy of LAMTOR2. (DOC) [file pone.0053768.s001.doc]

**Supplementary Figure S2** Amplification and sequencing strategy of *LAMTOR3*


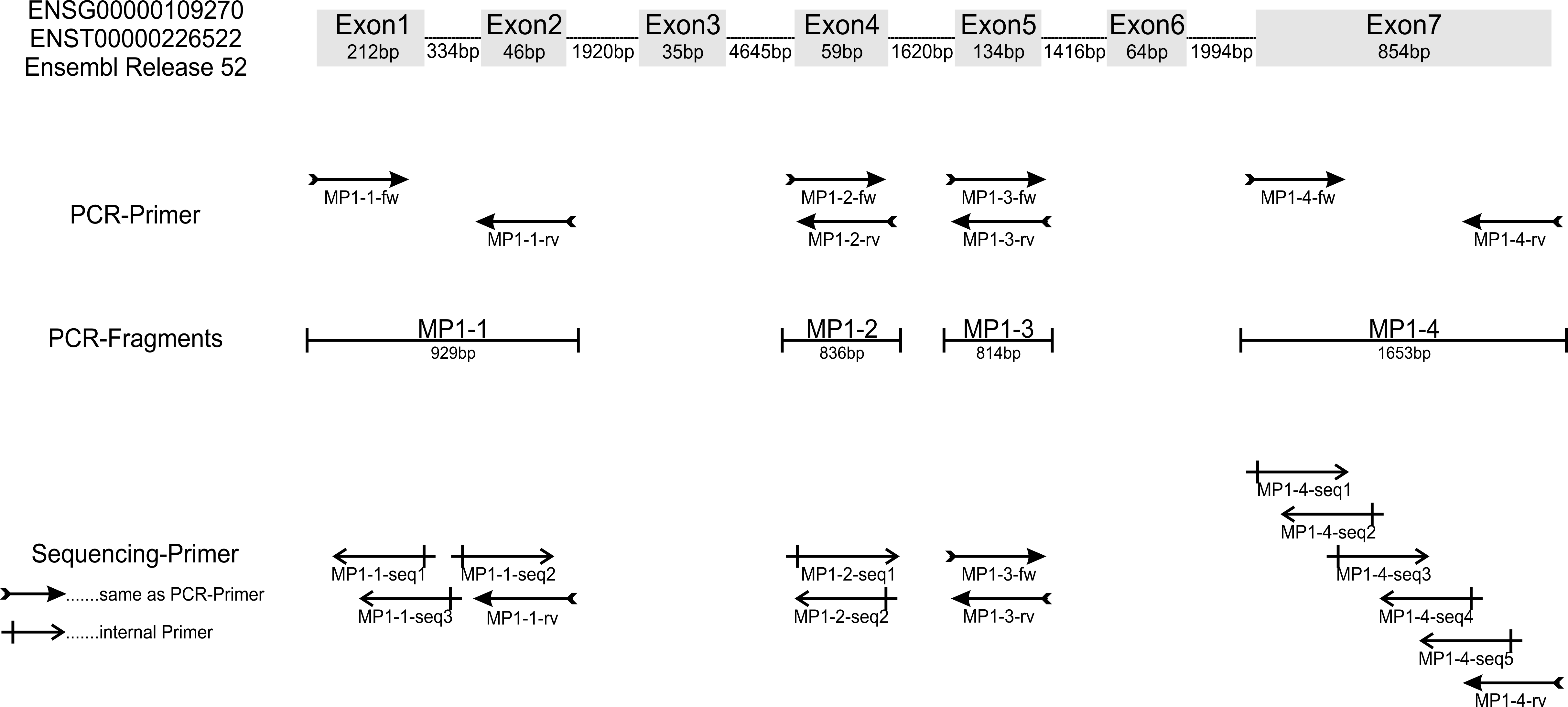

Supplement: Figure S2 — Amplification and sequencing strategy of LAMTOR3. (DOC) [file pone.0053768.s002.doc]
