# Supplementary material for: Polymorphisms in the Gene Regions of the Adaptor Complex LAMTOR2/LAMTOR3 and Their Association with Breast Cancer Risk
Source: PLoS One. 2013 Jan 16;8(1):e53768. doi: 10.1371/journal.pone.0053768 (PMC3547070; doi:10.1371/journal.pone.0053768)
Supplement: Table S1 — Primers used for amplification and sequencing of LAMTOR2. (DOC) [file pone.0053768.s003.doc]

**Supplementary Table S1** Primers used for amplification and sequencing of *LAMTOR2*

| **Primer** | **Function** | **Target** | **Sequence** |
| --- | --- | --- | --- |
| p14-1-fw | PCR & Seq | p14-1 | ggccacaaagaactacgattccc |
| p14-1-rv | PCR | p14-1 | ACCAGAACCCAAGTCAGACC |
| p14-1-seq2 | Sequencing | p14-1 | ACACCGGCTTCCCTTTTCGA |
| p14-1-seq1 | Sequencing | p14-1 | gggacacgctcaggccagag |
| p14-1-seq3 | Sequencing | p14-1 | TCCGGAGATCCTGTCCCTGC |
| p14-2-fw | PCR & Seq | p14-2 | ggtagggattacagaaagaa |
| p14-2-rv | PCR | p14-2 | TTTGCCTATGAGGTGGGTAA |
| p14-2-seq1 | Sequencing | p14-2 | taacatggttagtaggtggc |
| p14-2-seq2 | Sequencing | p14-2 | ccctgtttctagagttctca |
| p14-2-seq3 | Sequencing | p14-2 | TGAGGTCATGCCATTGGTGA |
| p14-2-seq4 | Sequencing | p14-2 | TCGCAGAAGGTCATGGTGAA |
