# Supplementary material for: Polymorphisms in the Gene Regions of the Adaptor Complex LAMTOR2/LAMTOR3 and Their Association with Breast Cancer Risk
Source: PLoS One. 2013 Jan 16;8(1):e53768. doi: 10.1371/journal.pone.0053768 (PMC3547070; doi:10.1371/journal.pone.0053768)
Supplement: Table S2 — Primers used for amplification and sequencing of LAMTOR3. (DOC) [file pone.0053768.s004.doc]

**Supplementary Table S2** Primers used for amplification and sequencing of *LAMTOR3*

| **Primer** | **Function** | **Target** | **Sequence** |
| --- | --- | --- | --- |
| MP1-1-fw | PCR | MP1-1 | ACATAACCTTGCTTGCATTT |
| MP1-1-rv | PCR & Seq | MP1-1 | CCTGCAAATTCATTCATATTAGTCT |
| MP1-1-seq1 | Sequencing | MP1-1 | AGAGCTAGGCCCAAAGAAAA |
| MP1-1-seq2 | Sequencing | MP1-1 | CTCTTCTTTTCTTTGGGCCTAG |
| MP1-1-seq3 | Sequencing | MP1-1 | CCGGTGACTCTTCCCTCTT |
| MP1-2-fw | PCR | MP1-2 | gctgataatctttcatgcttttgtg |
| MP1-2-rv | PCR | MP1-2 | ACTGACTCTCTGTGACATCATGTT |
| MP1-2-seq1 | Sequencing | MP1-2 | gtggtttttcagaaaataca |
| MP1-2-seq2 | Sequencing | MP1-2 | ATGATACATGTGATATATAATACA |
| MP1-3-fw | PCR & Seq | MP1-3 | actgctgctgtgggttttataag |
| MP1-3-rv | PCR & Seq | MP1-3 | AGTTGTTTAGCTAACGGCACCA |
| MP1-4-fw | PCR | MP1-4 | actttctgagcactaatatggcatt |
| MP1-4-rv | PCR & Seq | MP1-4 | ATGATGAGACATGAATGGTGCTTCT |
| MP1-4-seq1 | Sequencing | MP1-4 | ccagaattcaaaacacttctga |
| MP1-4-seq2 | Sequencing | MP1-4 | ACTGATCTATCTATAAATTACATCT |
| MP1-4-seq3 | Sequencing | MP1-4 | atagatagatcagttgctatatt |
| MP1-4-seq4 | Sequencing | MP1-4 | ATGTACAAAGGAAAGTTAGTGATTG |
| MP1-4-seq5 | Sequencing | MP1-4 | AGGTTTCTTTTCCAGTTAACAG |
